# Aminoglycoside-inducible expression of the *mexAB-oprM* multidrug efflux operon in *Pseudomonas aeruginosa*: Involvement of the envelope stress-responsive AmgRS two-component system

**DOI:** 10.1371/journal.pone.0205036

**Published:** 2018-10-05

**Authors:** Michael Fruci, Keith Poole

**Affiliations:** Department of Biomedical and Molecular Sciences, Botterell Hall, Queen’s University, Kingston, ON, Canada; Universite Paris-Sud, FRANCE

## Abstract

Exposure of *P*. *aeruginosa* to the aminoglycoside (AG) paromomycin (PAR) induced expression of the PA3720-*armR* locus and the *mexAB-oprM* multidrug efflux operon that AmgR controls, although PAR induction of *mexAB-oprM* was independent of *armR*. Multiple AGs promoted *mexAB-oprM* expression and this was lost in the absence of the *amgRS* locus encoding an aminoglycoside-activated envelope stress-responsive 2-component system (TCS). Purified AmgR bound to the *mexAB-oprM* promoter region consistent with this response regulator directly regulating expression of the efflux operon. The thiol-active reagent, diamide, which, like AGs, promotes protein aggregation and cytoplasmic membrane damage also promoted AmgRS-dependent *mexAB-oprM* expression, a clear indication that the MexAB-OprM efflux system is recruited in response to membrane perturbation and/or circumstances that lead to this. Despite the AG and diamide induction of *mexAB-oprM*, however, MexAB-OprM does not appear to contribute to resistance to these agents.

## Introduction

*Pseudomonas aeruginosa* is a common nosocomial human pathogen [1, 2] often associated with pulmonary infections in patients with cystic fibrosis [3]. The organism has an impressive intrinsic antimicrobial resistome [4] and readily develops resistance during antimicrobial therapy via mutation and horizontal gene transfer [5–7]. Major contributors to intrinsic and acquired antimicrobial resistance in *P*. *aeruginosa* are a number of broadly-specific multidrug efflux systems of the RND family [8]. One of these, MexAB-OprM, which contributes to both intrinsic [9] and acquired (i.e., mutational) [6] resistance, exhibits one of the broadest substrate profiles of the RND pumps in *P*. *aeruginosa*, accommodating a wide range of clinically-relevant [10–12] and experimental [13] antimicrobials as well as biocides [14] and a variety of non-clinical agents (e.g., organic solvents [15], dyes [16, 17], detergents [17], herbicides [18] and acylhomoserine lactones (AHLs) associated with quorum-sensing (QS) [19]. Clinically, this efflux system is most noted for its contribution to acquired fluoroquinolone and β-lactam resistance [6].

Expression of *mexAB-oprM* is controlled by several regulators including two repressors, MexR [20] and NalD [21], that each act at one of two promoters occurring distal (PI, MexR) [22] and proximal (PII, NalD) [21] to the efflux locus. Mutations in each of these genes are associated with efflux gene hyperexpression and multidrug resistance in both lab [20] [23] [24, 25] and clinical [26] [25, 27, 28] isolates. MexR binding to its promoter is modulated by its redox status (in response to oxidative stress) [29, 30] and by the ArmR anti-repressor, whose binding to MexR abrogates promoter binding by the repressor [31]. *armR* occurs as part of the PA3720-*armR* operon that is regulated by the product of the divergently-transcribed *nalC* repressor gene [32], with *nalC* mutants showing elevated PA3720-*armR* expression and, so, elevated *mexAB-oprM* expression and multidrug resistance [32] as a result of ArmR modulation of MexR's repressor activity [31]. The function of PA3720, a protein with no homology to any characterised protein, remains unknown. *nalC* lab and clinical isolates expressing *mexAB-oprM* and showing a multidrug-resistant phenotype have been reported [32] [33, 34]. Recently, a homologue of the *E*. *coli* CpxR envelope stress response regulator has been identified in *P*. *aeruginosa* and shown to bind to the PI promoter of *mexAB-oprM*, where it positively influences efflux gene expression [35]. Interestingly, *mexAB-oprM* expression and the multidrug resistance of MexR^-^ *nalB* mutants were shown to be partially dependent on CpxR [35]. Finally, the BrlR regulator of antimicrobial tolerance of *P*. *aeruginosa* biofilms [36] has also been shown to bind to the *mexAB-oprM* promoter region where it positively influences expression of the efflux operon, which thus contributes to biofilm antimicrobial tolerance [37].

Despite the identification of a number of *mexAB-oprM* regulators, the naturally-occurring signals to which they respond and the intended substrates and, so, function of the efflux system remain largely unknown. Chlorinated phenols, including the pesticide pentachlorophenol (PCP) that is an uncoupler of oxidative phosphorylation [38], have been shown to induce expression of the PA3720-*armR* and *mexAB-oprM* operons, in part owing to their ability to bind and, so, modulate the repressor activity of NalC [39, 40]. Still, some PCP induction of *mexAB-oprM* is seen in mutants lacking the ArmR anti-repressor but dependent on MexR, an indication that this agent operates though multiple channels in driving expression of this efflux operon. In any case, it seems unlikely that PCP is the intended natural substrate/inducer but may mimic naturally-occurring plant-derived phenolic compounds that could be. NalD has been shown to bind novobiocin, a known MexAB-OprM substrate, with novobiocin binding abrogating NalD interaction with its DNA target, thereby enhancing PII promoter activity [41]. Still, it is far from clear that this gyrase-targeting [42, 43] antimicrobial is an intended inducer and substrate for this system. The identification of a CpxR homologue as a regulator of *mexAB-oprM* would suggest that the efflux system responds to envelope stress. Still, an envelope stress-response two-component system (TCS) that regulates genes reminiscent of *E*. *coli* CpxR targets, AmgRS, has already been described in *P*. *aeruginosa*, despite AmgRS being a homologue of the *E*. *coli* osmotic stress-responsive TCS, OmpR-EnvZ [44]. As such, *P*. *aeruginosa* CpxR may respond to signals other than those related to envelope perturbation. Finally, a recent report documents a transient, early log phase induction of *mexAB-oprM* in response to Ca^2+^ [45], although the regulator mediating this and the Ca^2+-^generated signal(s) to which the system is responding have not been identified.

The current study was undertaken to follow up a preliminary observation that exposure of *P*. *aeruginosa* to the aminoglycoside (AG), PAR, induced the PA3720-*armR* operon, which suggested that this antimicrobial might also upregulate *mexAB-oprM* expression. We confirm that it and, in fact, several AGs, do indeed induce *mexAB-oprM* expression. Surprisingly, however, this was independent of the ArmR anti-repressor and instead dependent on the aforementioned AmgRS envelope stress-responsive TCS. These results suggest that the generation of certain product(s) of envelope stress and/or cellular conditions that promote envelope perturbation require MexAB-OprM function and, so, promote it’s production.

## Materials and methods

### Bacterial strains and plasmids

Bacterial strains and plasmids used in this study are listed in Table 1. Plasmid pEX18Tc and its derivatives were maintained in *E*. *coli* with 5 (in L-broth) or 10 (on L-agar) μg/ml tetracycline. Plasmid pET23a and its derivatives were maintained in *E*. *coli* with ampicillin (100 μg/ml).

**Table 1:** Bacterial strains and plasmids.

Strain	Relevant genotype^a^	Source
***E*. *coli***		
DH5α	φ80Δ*lacZ*ΔM15 *endA1 recA1 hsdR17*(r_K_^-^m_K_^+^) s*upE44 thi-1 gyrA96 relA1* F^-^ Δ(*lacZYA-argF*) *U169*	[46]
S17-1	*thi pro hsdR recA Tra*^+^	[47]
*E*. *coli* Bl21 DE3 (pLysE)	F^-^*ompT*r_B_^-^ m_B_^-^; DE3 is a lambda derivative carrying *lacI* and a T7 RNA polymerase gene under p*lac*UV5 control	[48]
***P*. *aeruginosa***		
K767	PAO1 wild-type *P*. *aeruginosa*	[49]
K1491	K767Δ*mexR*	[50]
K3415	K767Δ*armR*	[39]
K1523	K767Δ*mexB*	[51]
K1525	K767Δ*mexXY*	[52]
K1542	K767Δ*mexXY* Δ*mexB*	[53]
K3793	K767Δ*nalD*	This study
K3794	K767Δ*mexR* Δ*nalD*	This study
K3519	K767Δ*amgR*	[54]
K3583	K767Δ*amgS*	[54]
K3260	K767 derivative carrying the *amgS*_V121G_ mutation	[54]
K3249	K767 derivative carrying the *amgS*_R182C_ mutation	[54]
**Plasmids**		
pET23a	Expression plasmid; Ap^R^	Novagen
pMJF34	pET23a::*amgR*	This study

^a^Tc^R^, tetracycline resistance;

Ap^R^, ampicillin resistance.

### DNA methods

Standard protocols were used for restriction endonuclease digestions, ligations, transformations and agarose gel electrophoresis, as previously described [55]. Plasmid DNA was extracted from *E*. *coli* using the Fermentas GeneJET Plasmid Miniprep Kit or the Qiagen Plasmid Midi Kit according to protocols provided by the manufacturers. Chromosomal DNA was extracted from *P*. *aeruginosa* using the Qiagen DNeasy Blood & Tissue Kit according to a protocol provided by the manufacturer. PCR products and restriction endonuclease digest products requiring purification were purified using the Promega Wizard SV Gel and PCR Clean-Up System according to a protocol provided by the manufacturer. Plasmid DNA was introduced into CaCl_2_-competent *E*. *coli* cells, which were prepared as previously described [55]. Oligonucleotide synthesis was performed by Integrated DNA Technologies (Coralville, Iowa), and nucleotide sequencing was performed by ACGT Corporation (Toronto, Canada).

### Susceptibility testing

The antimicrobial susceptibility of various *P*. *aeruginosa* strains was assessed using the 2-fold microtiter broth dilution method as described previously [56]. The minimum inhibitory concentration (MIC) was recorded as the lowest concentration of antibiotic inhibiting visible growth after 18 hours of incubation at 37°C.

### Quantitative RT-PCR

*P*. *aeruginosa* cells grown overnight in L-broth at 37°C were subcultured (1:49) in the same medium and incubated at 37°C until cultures reached an optical density at 600 nm (OD_600_) of 0.6–0.8. Total RNA was isolated, purified and reverse transcribed into cDNA as described previously [57]. Where specified, the antimicrobials paromomycin (PAR; 256 μg/ml), neomycin (NEO; 64 μg/ml), gentamicin (GEN; 2 μg/ml), tobramycin (TOB; 1 μg/ml), kanamycin (KAN; 128 μg/ml), chloramphenicol (CAM; 32 μg/ml), tetracycline (TET; 16 μg/ml), erythromycin (ERY; 512 μg/ml), azithromycin (AZI; 512 μg/ml) and diamide (DIA; 4 mM) were added at their respective MICs 30 minutes prior to harvesting cells. In some experiments, *P*. *aeruginosa* was pretreated with CAM (128 *μ*g/mL) for 15 min prior to the addition of neomycin at its MIC. The primers used in quantitative real-time PCR (qPCR) were designed to amplify gene fragments with lengths of 138 bp (*mexA*; Forward: CAGCAGCTCTACCAGATCG; Reverse: CGTACTGCTGCTTGCTCA), 123 bp (*armR*; Forward: CAACAAACCGTCCCGCAC; Reverse: GTAGAGGTCCCAGGCATTGC) and 188 bp (PA3720; Forward: GATGCCTTTCCCTTGGTCCA; Reverse: TCCTTGAGCCACAACACCAG). The *rpoD* reference gene was amplified as described previously [58]. The amplification efficiencies of the qRT-PCR primer sets were 102.8% (correlation co-efficient, r^2^ = 0.997) for *mexA*, 100.3% for *armR* (r^2^ = 0.997) and 101.2% for PA3720 (r^2^ = 0.994). The qRT-PCR reaction mixtures, amplification parameters, and melt curve analyses were performed as previously described [39, 59]. The expression levels of *mexA*, PA3720 and *armR* were normalized to that of the reference gene *rpoD* using the ΔΔC(t) method provided by the CFX-manager software version 1.6 (Bio-Rad) and are reported as fold change relative to that in the wild-type *P*. *aeruginosa* PAO1 strain K767, unless otherwise specified. A minimum of three biological replicates each performed in triplicate were carried out for all samples. In all instances, no-template controls were carried out to ensure the absence of DNA contamination.

### Expression and purification of AmgR

To facilitate the purification of AmgR, a C-terminal polyhistidine tag was engineered onto AmgR by cloning the *amgR* gene into plasmid pET23a. The *amgR* gene was amplified by PCR using primers AmgR-His-For (5’-GATCCATATGTCGAACCCTGCCGCCCT-3’; NdeI site underlined) and AmgR-His-Rev (5’-GATCCTCGAGGGCCTTGCGCGCGTTGCCGTC-3’; XhoI site underlined) in a 50 μl PCR mixture that contained 1 μg *P*. *aeruginosa* PAO1 strain K767 chromosomal DNA, 0.6 μM of each primer, 0.2 mM of each dNTP, 1 x Phusion GC buffer and 1 U of Phusion polymerase (New England Biolabs). The mixture was heated for 30 sec at 98°C, followed by 30 cycles of 30 sec at 98°C, 30 sec at 65.0°C, 30 sec at 72°C, concluding with 7 min at 72°C. The PCR product was gel-purified, digested with NdeI and XhoI and cloned into NdeI-XhoI-restricted pET23a to yield pET23a::*amgR* (pMJF34) encoding His-tagged AmgR. Following nucleotide sequencing of the cloned gene to confirm the absence of PCR-generated mutations, plasmid pMJF34 was introduced into *E*. *coli* Bl21(DE3) harbouring the pLysE plasmid via transformation. AmgR-His was then expressed and purified from 50 ml of culture as previously described [39], with the exception that IPTG-induced expression of the *amgR* gene on pMJF34 was carried out for 90 min.

### Electrophoretic mobility shift assay

The binding of purified AmgR to a DNA fragment encompassing the intergenic region upstream of *mexAB-oprM* was assessed using the electrophoretic mobility shift assay (EMSA) as described previously [39]. The intergenic region was amplified on a 315-bp fragment using primers K9 and K10 [22] in a 50-μl reaction mixture containing 1 μg of purified *P*. *aeruginosa* PAO1 strain K767 chromosomal DNA, 0.6 μM of each primer, 0.2 mM of each dNTP, 1 x Phusion HF buffer, 5% (vol/vol) dimethylsulphoxide and 1 U of Phusion polymerase (New England Biolabs). The mixture was heated at 98°C for 30 sec, followed by 30 cycles of 30 sec at 98°C, 30 sec at 65.0°C, 15 sec at 72°C, concluding with 7 min at 72°C. The DNA fragment was gel purified and quantified using a Nanodrop^TM^ 2000 Spectrophotometer (Thermo Fisher Scientific). To assess the specificity of any binding observed, excess sheared salmon sperm DNA (100 μM) was added to the reaction mixtures prior to the addition of AmgR.

### Membrane depolarization assay

A previously described fluorometric assay [60] involving the membrane potential-sensitive dye bis-(1,3-dibutylbarbituric acid) trimethine oxonol [DiBAC4(3)], was employed to measure the degree of cytoplasmic membrane (CM) depolarization promoted by diamide treatment of *P*. *aeruginosa*. Briefly, early log phase (OD_600_ = 0.3–0.5) L-broth subcultures of *P*. *aeruginosa* were exposed (or not) to diamide (4 mM final concentration). Samples (5 mL) were taken immediately and then hourly over 3 h and incubated with DiBAC4(3) (Invitrogen, Burlington, Ontario, Canada; 10 μg/ml final concentration) in the dark for 5 min at 37°C. Bacteria were then pelleted and resuspended in phosphate-buffered saline [61] to a final OD_600_ of 0.1. The membrane depolarization-dependent fluorescence emitted by cells was measured using a Varian (now Agilent, Mississauga, Ontario, Canada) Cary Eclipse fluorescent spectrophotometer with excitation and emission wavelengths of 490 and 518, respectively.

## Results

### Aminoglycoside-induced expression of *mexAB-oprM*

**Fig 1:**
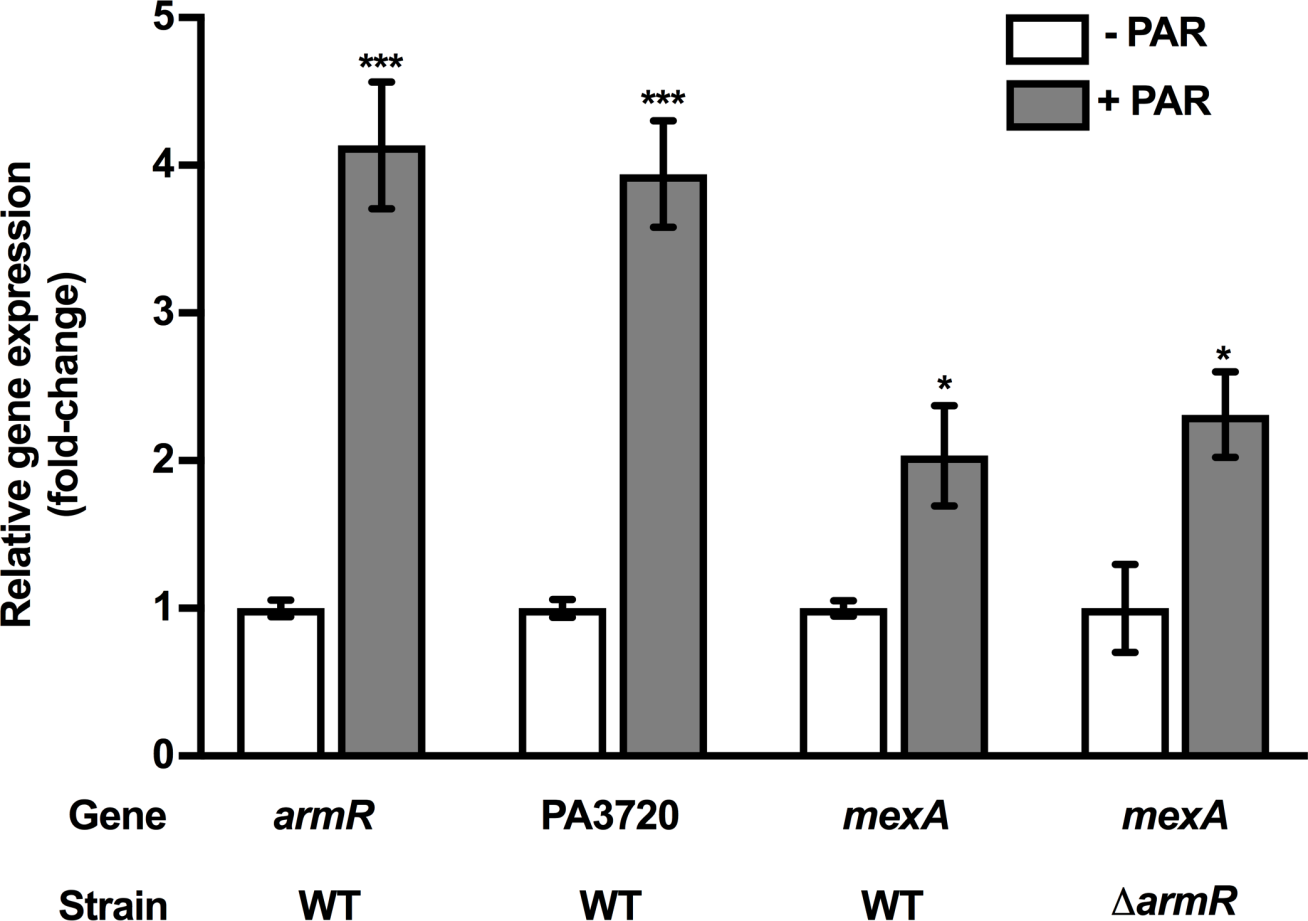
PAR induction of PA3720-*armR* and *mexAB-oprM* expression. Expression of PA3720, *armR* and *mexA* was assessed in log-phase WT *P*. *aeruginosa* strain K767 following a 30-minute exposure to the MIC of the AG, paromomycin (PAR; 256 μg/ml) using qRT-PCR. *mexA* expression was also assessed in a Δ*armR* derivative of K767, K3415, following exposure (30 min) to the MIC of PAR (256 μg/ml) using qRT-PCR. In all cases, expression was normalized to *rpoD* and is reported relative to the untreated WT *P*. *aeruginosa* strain K767 (fold-change). Values shown are means ± standard errors of the means (SEMs) from at least three independent determinations, each performed in triplicate. t-test: *, P < 0.05; ***, P < 0.001.

DNA microarray analysis of wild-type (WT) *P*. *aeruginosa* PAO1 strain K767 treated with the AG, PAR, revealed an increase in expression of the PA3720-*armR* two-gene operon (K. Poole, unpublished) whose products regulate expression of the *mexAB-oprM* multidrug efflux operon [31, 32]. To validate the results of the transcriptome analysis, log-phase K767 cells were similarly treated with the MIC of PAR, and expression of the PA3720 and *armR* genes were assessed using qRT-PCR. In agreement with the microarray data, treatment with PAR led to an increase (~4-fold) in expression of the PA3720 and *armR* genes (Fig 1). ArmR is an anti-repressor for the *mexAB-oprM* repressor [31], MexR, and its expression in so-called *nalC* mutants is responsible for the elevated *mexAB-oprM* expression and multidrug resistance of these mutants [32]. It was, therefore, hypothesized that the PAR-promoted increase in *armR* expression and, ultimately, ArmR production, would also drive derepression of the *mexAB-oprM* operon. Indeed, PAR provided for a ~2-fold increase in *mexA* gene expression (as a measure of *mexAB-oprM* expression) relative to the untreated K767 strain (Fig 1). Surprisingly, however, *mexAB-oprM* expression was still PAR-inducible in a mutant lacking *armR* (Fig 1), an indication that PAR induction of *mexAB-oprM* expression was not mediated by ArmR.

**Fig 2:**
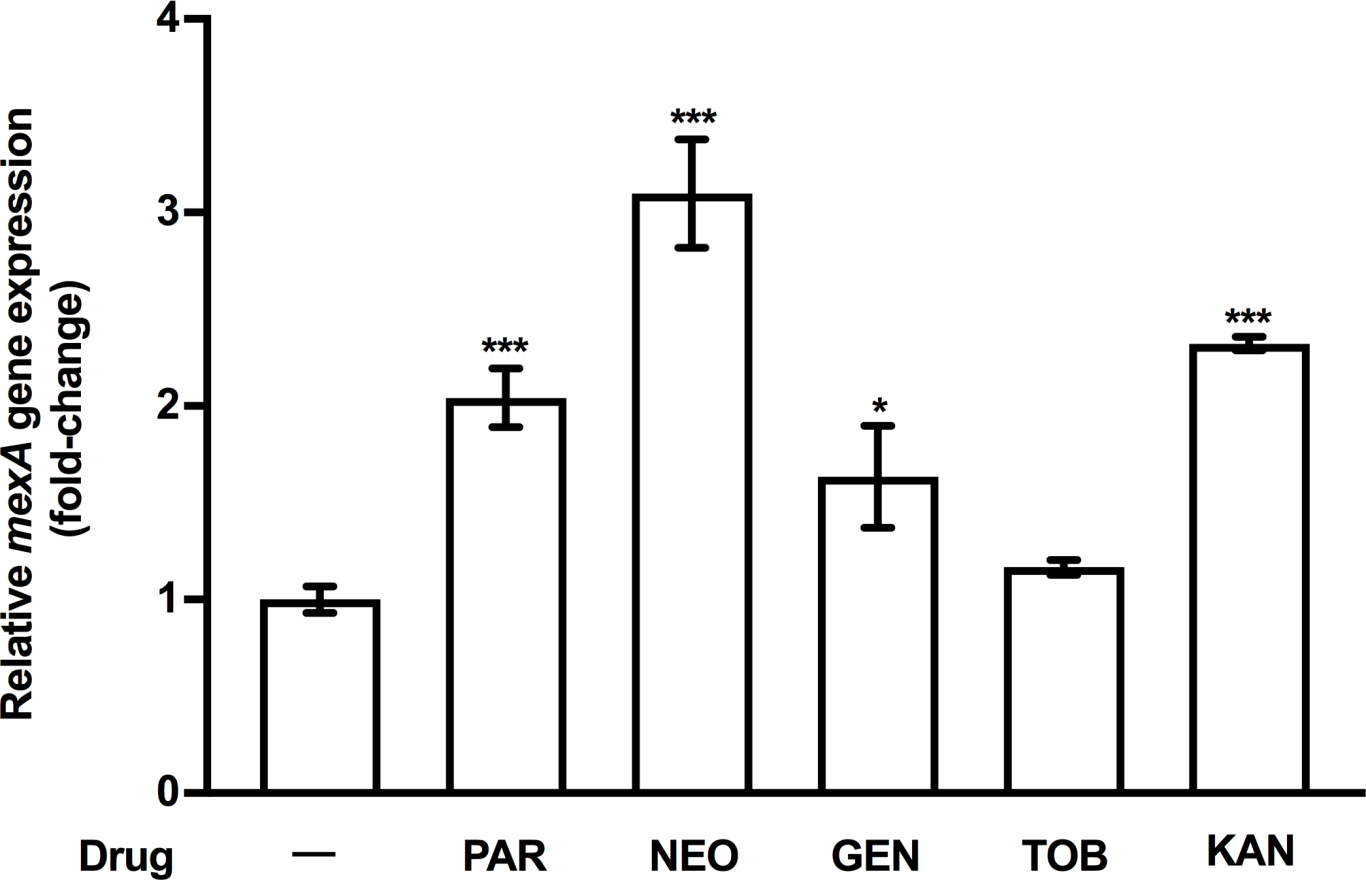
AG induction of *mexAB-oprM* expression. *mexA* expression was assessed in log-phase WT *P*. *aeruginosa* strain K767 following a 30-minute exposure to the MIC of the AGs, paromomycin (PAR; 256 μg/ml), neomycin (NEO; 64 μg/ml), gentamicin (GEN; 2 μg/ml), tobramycin (TOB; 1 μg/ml), and kanamycin (KAN; 128 μg/ml) using qRT-PCR. Expression was normalized to *rpoD* and is reported relative to the untreated K767 (fold change). Values are means ± SEMs from at least three independent determinations, each performed in triplicate. t-test: *, P < 0.05; ***, P < 0.001.

To assess whether PAR induction of this efflux system was specific to this AG or a more general property of this antimicrobial class, additional AGs were assessed for an ability to induce *mexAB-oprM* expression. Like PAR, the AGs KAN and NEO induced *mexAB-oprM* expression at their MICs, with NEO showing the best induction amongst all of the AGs tested (Fig 2). In contrast, GEN provided for a minimal increase in *mexAB-oprM* expression and TOB failed to induce this efflux operon, also at their MICs (Fig 2). Despite the induction of *mexAB-oprM*, however, this efflux system does not appear to contribute to AG resistance in *P*. *aeruginosa*–loss of MexAB-OprM in both WT strain K767 and a mutant derivative lacking the MexXY efflux system linked to intrinsic AG resistance did not impact AG susceptibility (Table 2).

**Table 2:** Impact of *mexAB-oprM* loss on AG susceptibility.

Strain	Genotype	MIC (μg/ml) for:^a^			
		GEN	PAR	NEO	KAN
K767	WT^b^	4	256	64	128
K1523	Δ*mexB*	4	256	64	128
K1525	Δ*mexXY*	2	32	32	64
K1542	Δ*mexXY* Δ*mexB*	2	32	32	64

^a^GEN, gentamicin; PAR, paromomycin; NEO, neomycin; KAN, kanamycin.

^b^WT, wild type

### AG induction of the *mexAB-oprM* operon is independent of the MexR and NalD repressors

**Fig 3:**
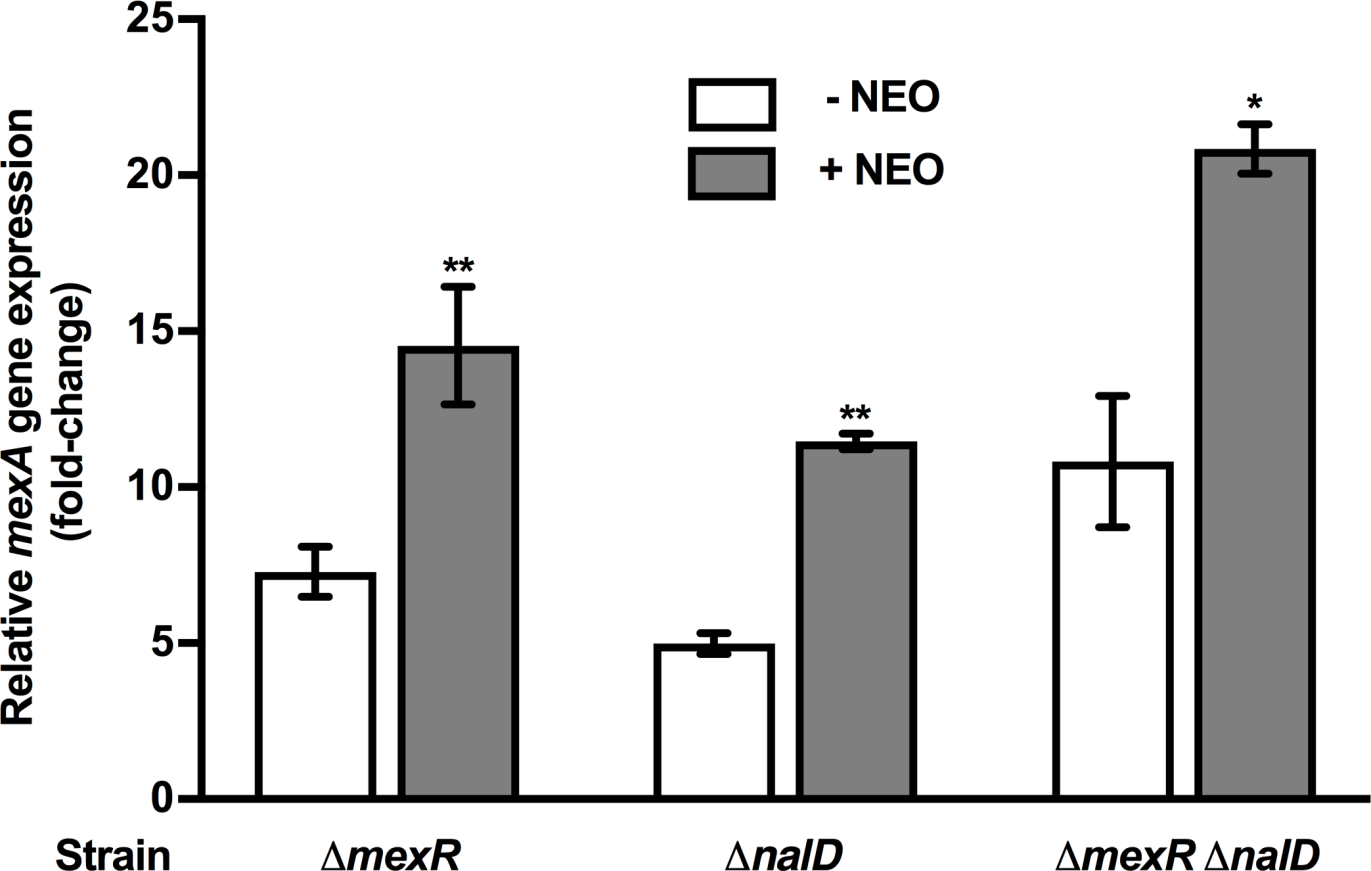
MexR- and NalD-independent AG induction of *mexAB-oprM* expression. *mexA* expression was assessed in *P*. *aeruginosa* strains K1491 (Δ*mexR*), K3793 (Δ*nalD*) and K3794 (Δ*mexR* Δ*nalD*) following a 30-minute exposure to the MIC of neomycin (NEO; 64 μg/ml for all strains) using qRT-PCR. Expression was normalized to *rpoD* and is reported relative to the untreated K767 strain (fold change). Values are means ± SEMs (error bars) from at least three independent determinations, each performed in triplicate. t-test: *, P < 0.05; **, P < 0.01.

The *mexAB-oprM* operon is regulated by 2 direct repressors, MexR [22] and NalD [21], one or both of which might mediate the observed AG inducibility of this efflux operon. To assess this, the impact of a *mexR* or *nalD* deletion on AG induction of *mexAB-oprM* expression was assessed. If AGs somehow obviate MexR or NalD repressor activity in promoting increased *mexAB-oprM* expression, AG induction of *mexAB-oprM* expression should be lost in either a *mexR* or *nalD* knockout strain. Individual deletions of the *mexR* and *nalD* genes in the WT K767 strain led to the expected increase in *mexAB-oprM* expression (Fig 3). Still, exposure of these mutants to the AG, NEO, still produced an increase in *mexAB-oprM* expression (Fig 3). Similar results were obtained with a mutant lacking both repressor genes, where loss of both *mexR* and *nalD* provided for an increase in *mexAB-oprM* expression that was, nonetheless, elevated further upon exposure to NEO (Fig 3). Thus, AG induction of this efflux operon is independent of the MexR and NalD repressors.

### AmgRS mediates AG induction of *mexAB-oprM* expression

**Fig 4:**
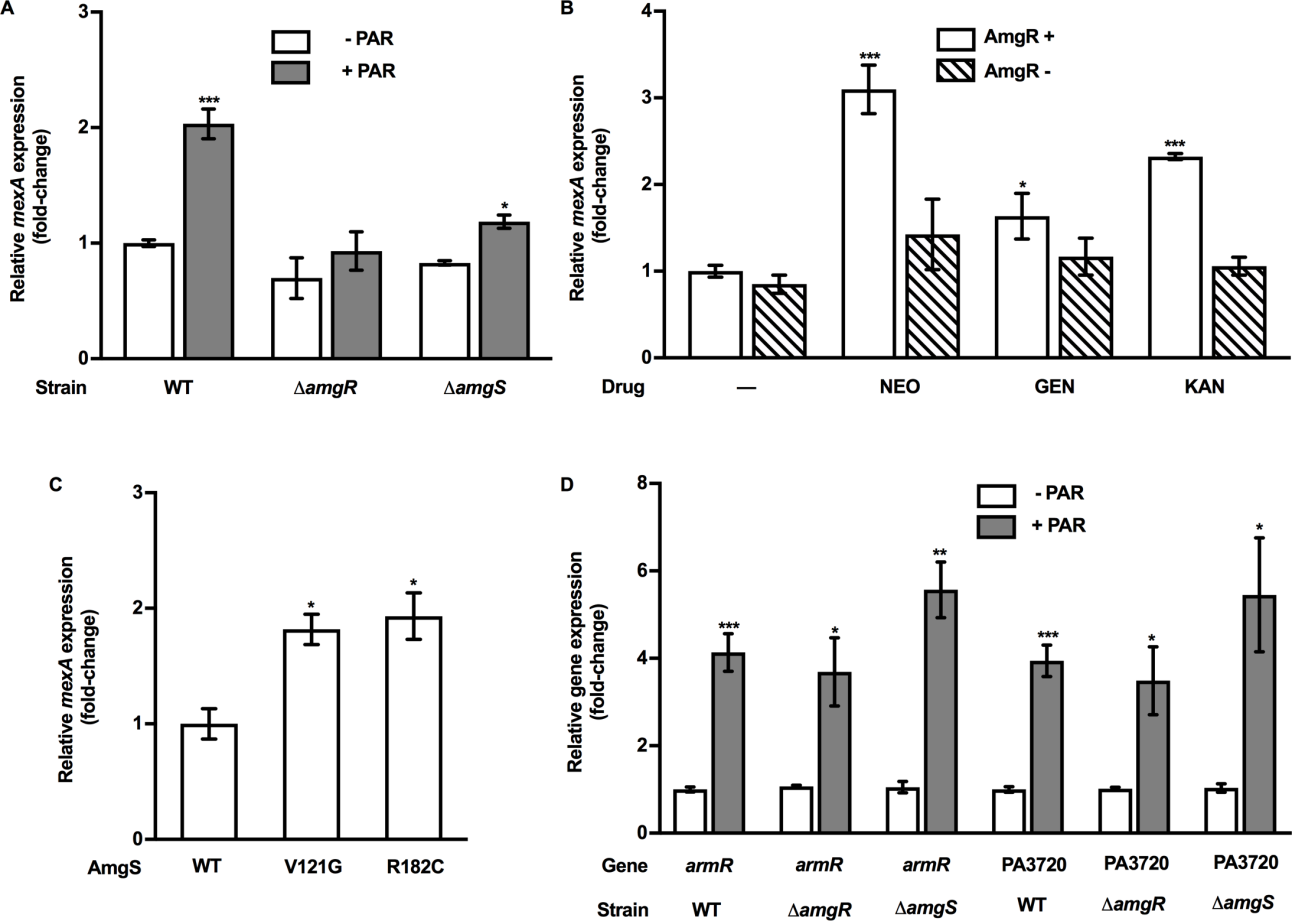
AmgRS-dependent AG-inducible *mexAB-oprM* expression. (**A**) *mexA* expression was assessed in log-phase cultures of WT *P*. *aeruginosa* strain K767 and its Δ*amgR* (strain K3159) and Δ*amgS* (strain K3583) derivatives following exposure to the MIC of PAR (K767, 256 μg/ml; K3159, 16 μg/ml; K3583, 16 μg/ml) for 30 min using qRT-PCR. (**B***) mexA* expression was assessed in log-phase cultures of *P*. *aeruginosa* strains K767 (WT; AmgR^+^) and K3159 (Δ*amgR*; AmgR^-^) exposed to the MIC of the indicated antimicrobials (-, no antimicrobial; NEO, neomycin; GEN, gentamicin; KAN; kanamycin) using qRT-PCR. Antimicrobials were used at: NEO, 64 (K767) and 4 (K3159) μg/ml; GEN, 2 (K767) and 0.5 (K3159) μg/ml; KAN, 128 (K767) and 16 (K3159) μg/ml. (**C**) *mexA* expression was assessed in strains harbouring WT (K767) and mutant (K3288, AmgS_V121G;_ K3249, AmgS_R182C_) *amgS* genes using qRT-PCR. (**D**) *armR* and PA3720 expression was assessed in log-phase cultures of *P*. *aeruginosa* strains K767 (WT), K3159 (Δ*amgR*), and K3583 (Δ*amgS*) exposed to the MIC of PAR for 30 min using qRT-PCR. Expression in all cases was normalized to *rpoD* and is reported relative to the untreated K767 strain (fold change). Values are means ± SEMs from at least three independent determinations, each performed in triplicate. t-test: *, P < 0.05; **, P < 0.01; ***, P < 0.001.

AmgRS, an AG-responsive TCS in *P*. *aeruginosa* that functions as part of an envelope stress response to AG-induced CM-damaging aberrant polypeptides, was recently shown to play a role in AG-induced expression of the AG resistance-promoting *mexXY* multidrug efflux operon [44, 54, 62]. This TCS might, thus, mediate the AG induction of *mexAB-oprM*. To assess this, the impact of AmgRS loss on PAR induction of *mexAB-oprM* expression was determined. As seen in Fig 4A, mutants lacking *amgR* or *amgS* were deficient for PAR-inducible expression of *mexAB-oprM*, an indication that AG induction of this efflux system was dependent on the AmgRS TCS. Consistent with this, *mexAB-oprM* expression promoted by the other AGs was similarly lost in the *amgR* deletion strain (Fig 4B). As expected, activation of AmgRS as a result of *amgS* gain-of-function mutations (V121G and R182C) also promoted an increase in *mexAB-oprM* expression, independent of AG exposure (Fig 4C). This did not, however, parallel an increase in resistance to representative MexAB-OprM substrate antimicrobials (e.g. carbenicillin, chloramphenicol, nalidixic acid; Table 3). As well, despite its link to *mexAB-oprM* and a common inducibility by PAR, the PA3720-*armR* operon retained its PAR inducibility in the *amgR* deletion strain (Fig 4D) an indication that these operons can be independently regulated.

**Table 3:** Impact of *amgS* gain-of-function mutations on antibiotic susceptibility.

Strain	AmgS^a^	MIC (μg/ml) for:^b^			
		CAR	CAM	NAL	NOV
K767	WT	64	64	128	1024
K3260	V121G	64	32	128	2048
K3249	R182C	64	64	128	1024

^a^The amino acid change in AmgS in the indicated mutants is highlighted. WT, wild type.

^b^CAR, carbenicillin; CAM, chloramphenicol; NAL, nalidixic acid; NOV, novobiocin.

### AmgR binds to the *mexAB-oprM* promoter region

**Fig 5:**
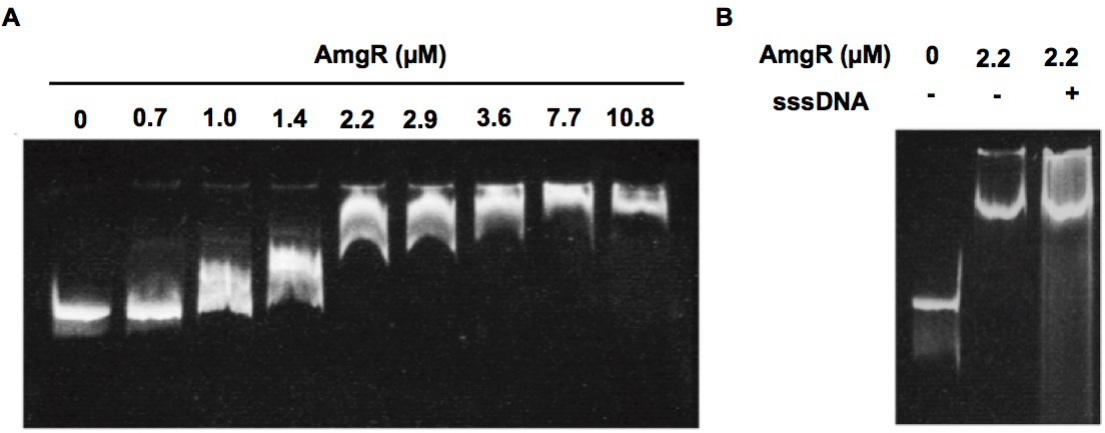
AmgR binds to the *mexAB-oprM* promoter region. (**A**) Electromobility shift assay in which 40 ng of a 351-bp DNA fragment encompassing the intergenic region upstream of *mexAB-oprM* was incubated with increasing concentrations of purified AmgR as indicated. (**B**) Electromobility shift assay in which the above-mentioned 351-bp DNA fragment was incubated with the indicated concentration of AmgR in the absence (-) and presence (+) of 200 ng of sheared salmon sperm DNA (sssDNA).

The regulation of *mexAB-oprM* by AmgRS is most simply explained by AmgR directly controlling expression of the efflux operon from a promoter upstream of the efflux genes. To assess this, binding of AmgR to the *mexAB-oprM* promoter region was assessed using an EMSA. As seen in Fig 5A, purified AmgR bound to a DNA fragment that encompassed the intergenic region upstream of *mexAB-oprM*. Moreover, binding was retained in the presence of excess competitor DNA (Fig 5B), an indication that binding was specific.

### Role of cytoplasmic membrane perturbation in AG-induced *mexAB-oprM* expression

**Fig 6:**
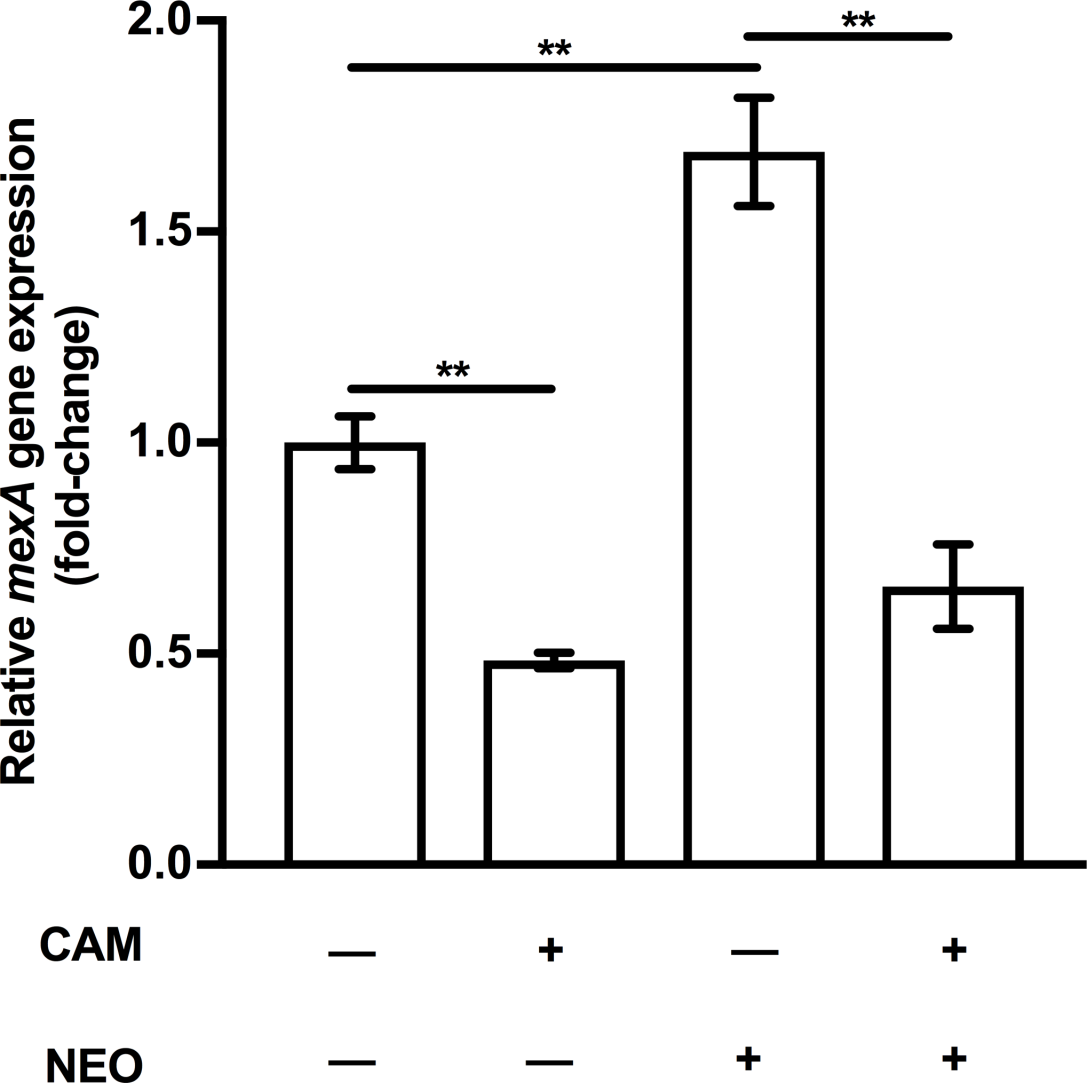
Impact of chloramphenicol on AG induction of *mexAB-oprM* expression. *mexA* expression was assessed in log phase cells of *P*. *aeruginosa* strain K767 exposed to NEO (at MIC; 64 μg/ml) for 30 min without (-) and with (+) a 15-min pre-treatment with chloramphenicol (CAM; 128 μg/ml) using qRT-PCR. In a control experiment, K767 was also exposed to 128 μg/ml CAM in the absence of NEO, to assess its impact on *mexA* expression on its own. Expression was normalized to *rpoD* and is reported relative to the untreated K767 strain (fold change). Values are means ± SEMs from at least three independent determinations, each performed in triplicate. t-test: **, P < 0.01.

It has been demonstrated that AmgRS responds to and protects cells from envelope stress caused by AG-generated mistranslated/misfolded proteins that damage the cytoplasmic membrane (CM) [44, 54, 62]. Previous reports demonstrated that treatment of WT *P*. *aeruginosa* with CAM, a protein translation inhibitor, prior to the addition of an AG, blocked AG-promoted CM damage, consistent with is blockage of mistranslated protein synthesis [62]. To assess if AG-promoted CM damage is responsible for AmgRS-dependent induction of *mexAB-oprM* expression by AGs, WT *P*. *aeruginosa* K767 cells were pre-treated with CAM prior to the addition of the AG, NEO, and *mexA* expression was measured. CAM pre-treatment of cells prevented NEO induction of *mexAB-oprM* expression (Fig 6), consistent with it blocking AG-generated mistranslated/misfolded proteins and subsequent CM damage. Still, it was also observed that CAM alone negatively influenced *mexAB-oprM* expression (Fig 6). As such, it is unclear whether AG-induced *mexAB-oprM* expression follows from AG generation of membrane-damaging mistranslation products.

### *mexAB-oprM* is inducible by additional ribosome-perturbing agents

**Fig 7:**
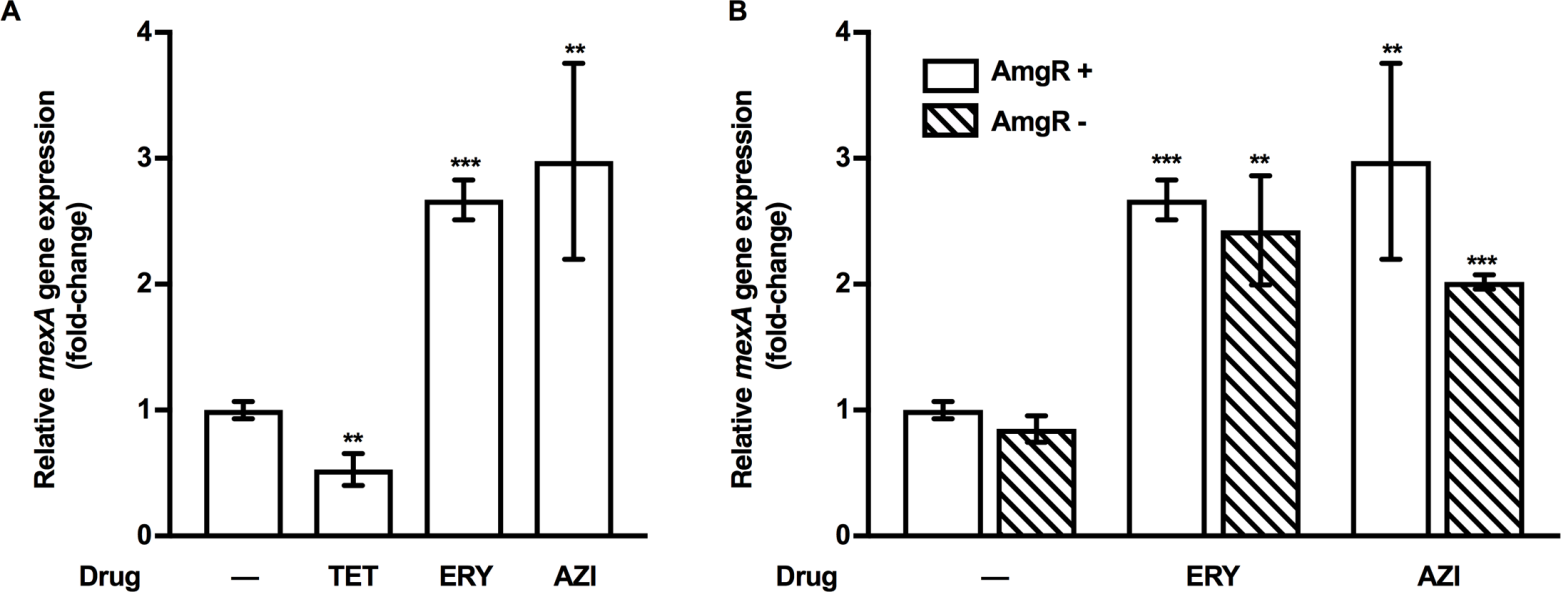
AmgRS-independent macrolide induction of *mexAB-oprM* expression. (**A**) *mexA* expression was assessed in log-phase cultures of WT *P*. *aeruginosa* strain K767 exposed to the MIC of tetracycline (TET; 16 μg/ml), erythromycin (ERY; 512 μg/ml) and azithromycin (AZI; 512 μg/ml) for 30 min using qRT-PCR. Expression was normalized to *rpoD* and is reported to that of the untreated (-) strain K767. (**B**) *mexA* expression was assessed in log-phase cultures of *P*. *aeruginosa* strains K767 (AmgR^+^) and K3159 (AmgR^-^) exposed to the MIC of ERY or AZI (512 μg/ml for both drugs and both strains) for 30 min using qRT-PCR. In all cases, expression was normalized to *rpoD* and is reported relative to that of the untreated strain K767 (fold change). Values are means ± SEMs from at least three independent determinations, each performed in triplicate. t-test: **, P < 0.01; ***, P < 0.001.

The observation that AG induction of *mexAB-oprM* is AmgRS-dependent and that CAM, another ribosome-targeting agent, fails to induce the efflux system, is consistent with AG-generated membrane-damaging mistranslation products being key to AG-promoted *mexAB-oprM* expression. As such, only mistranslation-promoting AGs are likely to induce this efflux system. To assess this, a number of additional agents that target the ribosome but do not promote mistranslation (TET, ERY, AZI) [63] were assessed for an ability to induce expression of the *mexAB-oprM* operon. As with CAM, treatment of WT *P*. *aeruginosa* K767 with the MIC of TET yielded a decrease in *mexAB-oprM* expression (2-fold; Fig 7A). In contrast, treatment of K767 with ERY or AZI increased *mexAB-oprM* gene expression (2.5-fold; Fig 7A). Still, this was independent of AmgR–induction of *mexAB-oprM* expression by ERY and AZI was retained in an *amgR* null mutant (Fig 7B; see K3519). Thus, ribosome perturbation alone is insufficient for AmgRS-promoted *mexAB-oprM* expression. While macrolide-induced *mexAB-oprM* expression is clearly mediated by a different regulatory pathway, it is unclear whether it follows from ribosome perturbation or some other impact of these agents on *P*. *aeruginosa*.

### Diamide-promoted *mexAB-oprM* expression

**Fig 8:**
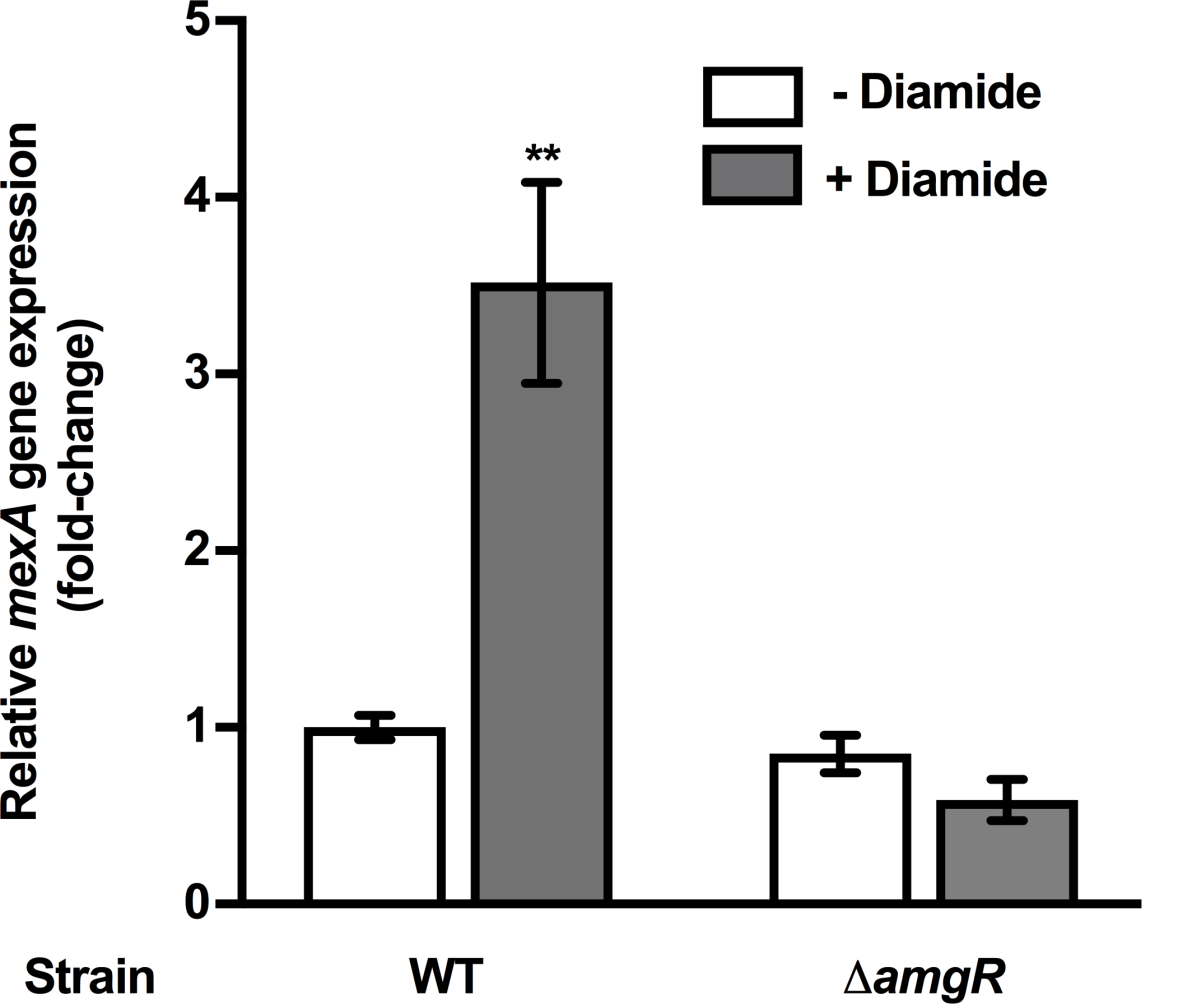
AmgRS-dependent diamide induction of *mexAB-oprM* expression. *mexA* expression was assessed in log-phase cultures of *P*. *aeruginosa* strains K767 (AmgR^+^) and K3159 (AmgR^-^) following a 30-min exposure to the MIC of diamide (4 mM for both strains) using qRT-PCR. Expression was normalized to *rpoD* and is reported to that of the untreated strain K767 (fold change). Values are means ± SEMs from at least three independent determinations, each performed in triplicate. t-test: **, P < 0.01.

**Fig 9:**
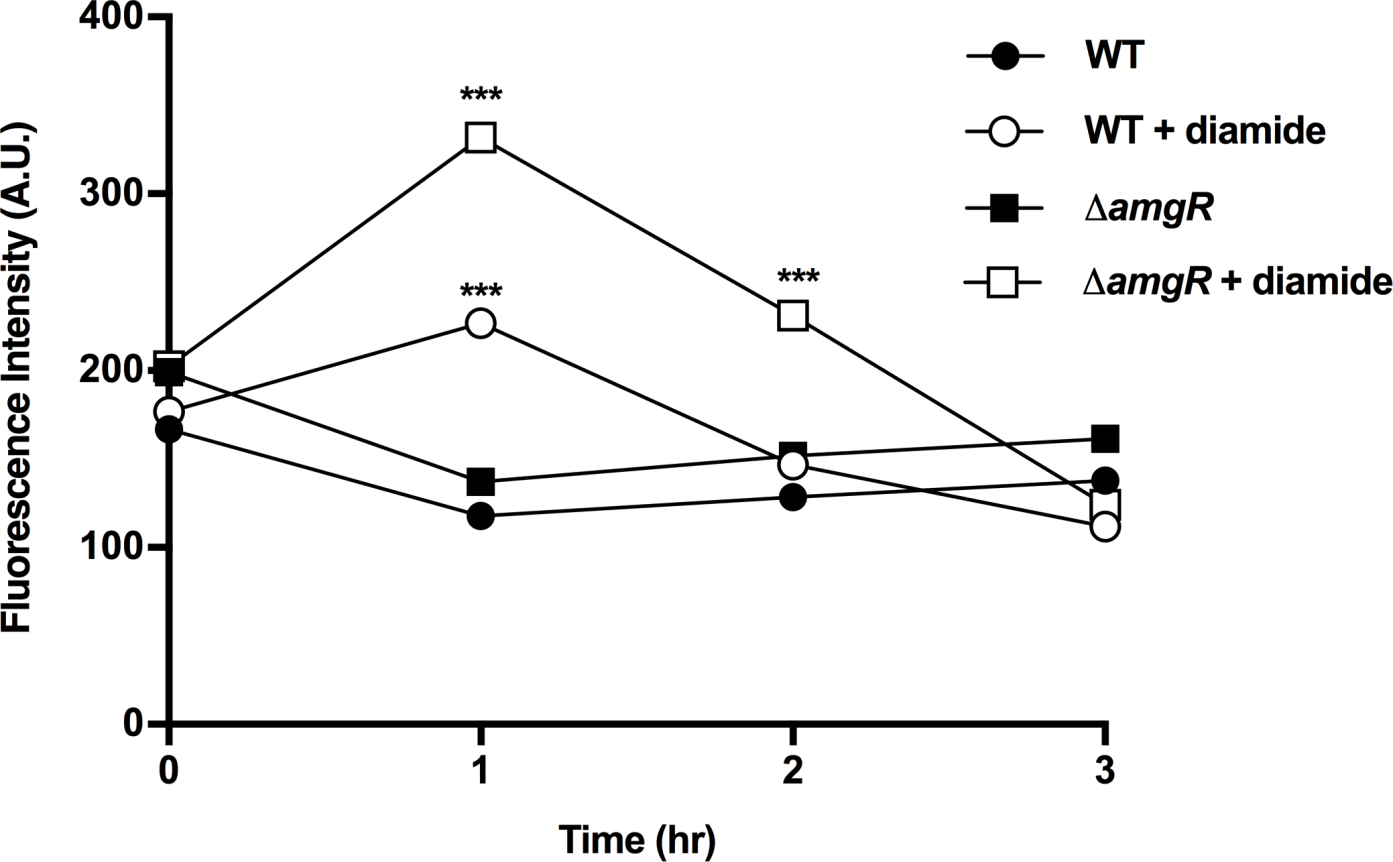
Diamide-promoted cytoplasmic membrane depolarization. Cytoplasmic membrane depolarization, as assessed by DiBAC4(3) fluorescence (arbitrary units; A.U.), was measured over time following exposure of WT strain *P*. *aeruginosa* K767 (circles) and its AmgR^-^ derivative, K3159 (squares), to 4 mM diamide (filled symbols) at T = 0 hr. Unexposed cells are represented by unfilled symbols. The data are means ± SEMs of three independent experiments. Note that the symbols are larger than the error bars, which are thus not visible in the figure. t-test: ***, P < 0.001.

In addition to their contribution to CM damage, AGs [e.g. streptomycin (STR)] have also been shown to cause protein aggregation in *E*. *coli*, also as a result of AG-induced mistranslation [64]. Like AGs, the thiol-specific oxidant, diamide (DIA) has also been shown to promote protein misfolding and aggregation [65–67] although an impact on membranes has never been assessed. To assess whether protein aggregation might be playing a role in AmgRS-driven *mexAB-oprM* expression the impact of diamide on expression of the efflux operon was assessed. Treatment of WT *P*. *aeruginosa* strain K767 with the MIC of diamide induced *mexAB-oprM* expression (~3.5-fold; Fig 8) and this was dependent on AmgR (Fig 8). Interestingly diamide was also shown to be modestly CM perturbing, promoting a transient increase in membrane depolarization (at 1 h post-diamide addition; Fig 9) and, as with AGs [60, 62], this was enhanced in a mutant lacking *amgR* (Fig 9). Thus, AmgRS appears to protect *P*. *aeruginosa* from CM perturbation promoted by diamide.

## Discussion

AmgR represents yet another direct regulator of the *mexAB-oprM* multidrug efflux operon, highlighting the complexity of the regulation of this locus and the apparent diversity of signals and growth conditions to which it responds. Given the apparent responsiveness of the AmgRS TCS to envelope stress resultant from cytoplasmic membrane perturbation by AG-generated mistranslation products, it is likely that the observed AmgRS-dependent induction of *mexAB-oprM* by AGs reflects an envelope stress-inducibility of this efflux locus and, so, a need for MexAB-OprM under certain conditions of envelope stress. Consistent with this, another agent shown here to be membrane perturbing, diamide, also showed AmgRS-dependent induction of *mexAB-oprM* expression and this TCS appeared to protect *P*. *aeruginosa* somewhat from diamide-mediated membrane damage. While CM perturbation is not a heretofore reported property of diamide, it has been shown that a mutant of *Xanthamonas campestris* pv. *campestris* lacking the RpoE envelope stress response sigma is more sensitive to diamide than WT *X*. *campestris* [68], further support for this agent being membrane-damaging. It is interesting to note, too, that the AG induction of *mexAB-oprM* was variable and reflected the AG responsiveness of AmgRS [54] with PAR and NEO being the better inducers/activators while, for example, TOB failed to induce/activate (at 1X MIC) and, indeed, perturb membranes [62]. Still, despite the induction of this multidrug efflux locus by AGs and diamide, MexAB-OprM does not appear to play any role in resistance to these agents. It is also possible that AG induction of *mexAB-oprM* serves primarily to increase OprM levels, this outer membrane protein functioning with the proteins products of the similarly AmgRS-regulated and AG-inducible *mexXY* efflux operon, with MexXY-OprM a known contributor to AG resistance. Still, *oprM* appears to possess its own promoter [69], such that its expression can be driven independently of *mexAB*, to ensure, for example, adequate levels of OprM to partner with MexXY without the need for unnecessary and wasteful induction of the *mexAB* genes.

While attempts were made to show that AG-promoted *mexAB-oprM* expression was dependent on protein translation (as evidence for potentially membrane-damaging mistranslation products being key to AG induction of the efflux locus) the results were inconclusive since classical translation inhibitors (CAM and TET) themselves had a negative impact on *mexAB-oprM* expression, rendering the observed CAM prevention of AG-promoted *mexAB-oprM* expression not readily interpretable. Possibly, TET and CAM inhibition of translation interferes with endogenous mistranslation that might provide basal levels of membrane-damaging aberrant polypeptides that activate AmgRS and provide for some *mexAB-oprM* expression. Still, loss of *amgR* did not adversely affect endogenous *mexAB-oprM* expression, arguing against this. Alternatively, since CAM is known to induce expression of a 2^nd^ RND type multidrug efflux operon, *mexEF-oprN* [70], and mutational upregulation of this efflux operon has been shown to parallel a possibly compensatory decrease in *mexAB-oprM* expression [71, 72], the CAM-driven decrease in *mexAB-oprM* expression may be secondary to its upregulation of *mexEF-oprN*. TET (and CAM) also induces the *mexXY* RND efflux operon [73] although there is as yet no evidence that this parallels a decrease in *mexAB-oprM* expression, and other *mexXY* inducers (AGs and macrolides) [73, 74] were shown here to promote *mexAB-oprM* expression. This may, however, simply reflect these ribosome-targeting agents impacting the *P*. *aeruginosa* in ways that require MexAB-OprM and, so, induce *mexAB-oprM* expression, masking the negative impact their possible induction of a 2^nd^ efflux operon might otherwise have on it.

Adding to the complexity of *mexAB-oprM* regulation is the observed induction of this efflux locus by the macrolide antibiotics ERY and AZI. That such induction is independent of AmgRS, consistent with this TCS responding to membrane perturbation and macrolides not known to provide for membrane damage, speaks to a possibly additional *mexAB-oprM* regulator mediating this, though at this point one cannot rule out the involvement of one of the other regulators identified to date. Interestingly, and in contrast to results presented here, it has been reported that AZI suppresses *mexAB-oprM* expression in *P*. *aeruginosa* [75], which we can only attribute to differences in growth medium and/or the WT strain used in the two studies. The observed AG induction of the PA3720-*armR* operon that encodes the MexR antirepressor is also interesting, given that it does not provide for AG induction of *mexAB-oprM* expression and, so, suggests that this locus may also influence expression of additional loci.

It is becoming clear that the *mexAB-oprM* multidrug efflux locus is inducible by a number of antimicrobial agents although, with few exceptions, the actual inducing signals and intended efflux substrates are unknown. Thus, while clearly stress-responsive, the actual function of MexAB-OprM remains to be elucidated.
